# Successful Treatment of Mucocutaneous Lupus Erythematosus in a Dog with Prednisolone, Mycophenolate Mofetil and Tacrolimus

**DOI:** 10.3390/vetsci8050072

**Published:** 2021-04-23

**Authors:** Jae-Eun Hyun, Yeong-Hun Kang, Cheol-Yong Hwang

**Affiliations:** 1 Department of Veterinary Internal Medicine, Konkuk Veterinary Medical Teaching Hospital, Konkuk University, Seoul 05029, Korea; 2 Laboratory of Veterinary Dermatology and the Research Institute for Veterinary Science, College of Veterinary Medicine, Seoul National University, Seoul 08826, Korea; yhkang92@snu.ac.kr (Y.-H.K.); cyhwang@snu.ac.kr (C.-Y.H.)

**Keywords:** mucocutaneous lupus erythematosus, dog, mycophenolate mofetil, prednisolone, tacrolimus

## Abstract

A 6-year-old, intact male miniature Pinscher dog had erosive lesions on perilabial, peripenial and perianal mucocutaneous areas, which were exacerbated by ulcerations, crusts, with pain while defecating and urinating. The lesions were symmetrical, and no systemic signs were observed. Histopathological evaluation showed parakeratotic hyperkeratosis, ulceration and cell-rich lymphoplasmacytic interface dermatitis with basal keratinocyte apoptosis. Immunohistochemistry revealed strong reaction in the dermoepidermal junction against goat-canine IgG and mild-to-moderate reaction against goat-canine IgA, IgM and C3. Based on these findings, the dog was diagnosed with mucocutaneous lupus erythematosus (MCLE). Oral prednisolone 1 mg/kg twice daily, mycophenolate mofetil (MMF) 18.3 mg/kg twice daily and 0.1% tacrolimus ointment were prescribed as initial treatment. The lesions showed remarkable improvement within 4 weeks, but the dog exhibited polyuria, polydipsia and hepatomegaly with high dosage of prednisolone. Hence, the dosage of prednisolone was gradually tapered for 9 weeks and discontinued, but MMF and tacrolimus were continued. No new lesion or associated side effect was observed while reducing the MMF dose to 10 mg/kg twice daily and with continuous use of tacrolimus ointment after steroid discontinuation. In conclusion, this case report emphasizes the usefulness of MMF and tacrolimus as steroid-sparing agents in the treatment of dogs with MCLE. To the best of our knowledge, this is the first case report of MCLE that was successfully managed long-term with MMF and tacrolimus.

## 1. Introduction

Among the major variants of the classification of human chronic cutaneous lupus erythematosus, mucosal involvement has been reported to occur only in patients with discoid lupus erythematosus (DLE), in especially few patients [1]. Similarly, a German Shepherd dog diagnosed with DLE was first reported to have perivulvar lesions in 1995 [2]. However, canine DLE is generally characterized by facial-predominant form, and juxtamucosal erosive lesions are not common [3]. Consequently, patients with DLE with such lesions were specifically referred to as having perianal or perivulvar lupus erythematosus, and the term mucocutaneous lupus erythematosus (MCLE) was proposed and its characteristic lesions and histological features were introduced in 2015 [4]. The most common clinical feature of MCLE in dogs is the development of mucocutaneous sores and pain that manifests during defecating (dyschezia) and/or urinating (dysuria). The lesions of MCLE generally present with symmetrical, well-demarcated erosions/ulcerations, erythema, crusts and hyperpigmentation involving the genital/perigenital or anal/perianal regions, perioral area, periocular skin and nasal planum or paranasal skin. Histopathological evaluation shows lymphocyte-rich interface dermatitis with basal keratinocyte damage, which is similar to ‘classic’ canine lupus erythematosus (CLE). IgG deposition at the basement membrane zone is generally found in immunohistochemistry.

In general, dogs with MCLE show good response to oral glucocorticoids, but they show relapses commonly after tapering or cessation of medication [4]. For long-term treatment, tetracycline/niacinamide may be beneficial; however, it is necessary to evaluate the efficacy of topical or other systemic immunosuppressive drugs. Mycophenolate mofetil (MMF) has been used for several years in veterinary medicine to treat immune-mediated inflammatory diseases, especially immune-mediated haemolytic anaemia, and its effects have also been reported in various skin diseases, including pemphigus foliaceus, vasculitis, subepidermal bullous disease, exfoliative cutaneous lupus erythematosus (ECLE) and vesicular cutaneous lupus erythematosus (VCLE) [5,6,7,8]. When compared with other adjunct immunosuppressive agents, MMF exhibits a rapid onset of action and tolerable side effects, based on which it can be suggested as a steroid-sparing agent for treating dogs with autoimmune or immune-mediated skin diseases [5,7]. The topical calcineurin inhibitor tacrolimus has been recognized as a relatively effective and safe drug for treating chronic inflammatory skin diseases, including CLE, in human medicine, and its therapeutic use has been reported for DLE and VCLE in dogs [9,10].

In this case report, we describe a case of MCLE in a miniature Pinscher dog in which effective improvement was achieved with initial systemic prednisolone, MMF and topical tacrolimus, followed by long-lasting complete remission with MMF and topical tacrolimus treatment.

## 2. Case History

A 6-year-old, intact male miniature Pinscher dog with a 4-month history of painful mucocutaneous ulcerative dermatitis with crusts was referred to our hospital. Past pertinent history included no improvement with 1 month’s administration of an anti-inflammatory dose (0.5 mg/kg orally twice daily) of oral prednisolone and application of antimicrobial ointment and antiseptic shampoo prescribed by a referring veterinarian. The dog lived indoors with another dog with no skin lesions and was generally healthy except for skin lesions and pain when defecating and urinating. The initial skin lesion was crusting lesions on the perilabial area, which became ulcerated and extended to the peripenial and perianal areas.

**Figure 1:**
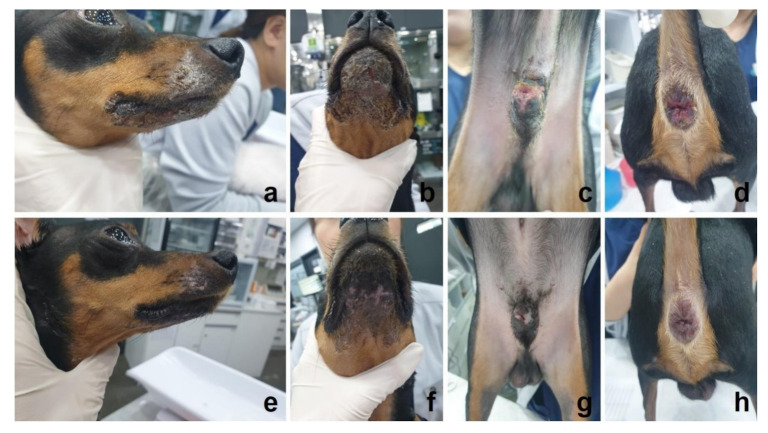
Mucocutaneous lupus erythematosus (MCLE) in a dog. Painful crusting erosive or ulcerative lesions on perilabial (**a**), muzzle (**b**), peripenial (**c**) and perianal (**d**) mucocutaneous junction on initial examination. Improved skin lesions (**e**–**h**) after one month of treatment with immunosuppressive dose of prednisolone, mycophenolate mofetil (MMF) and 0.1% topical tacrolimus treatment.

At presentation, the physical examination and thoracic radiography showed no specific finding. Abdominal radiology and ultrasonography revealed no other abnormalities except for mild hepatomegaly and vacuolar hepatopathy, which may be attributed to previous steroid administration. Mild elevated levels of globulin (4.8 g/dL; reference range 2.5–4.5 g/dL), blood urea nitrogen (32.7 mg/dL; reference range 20–30 mg/dL) and C-reactive protein (37 mg/L; reference range 1–9 mg/L) were found in the hemogram. Macroscopic examination revealed multifocal painful ulcerations and crusts on the perilabial area and symmetrical painful ulcerations in the peripenial and perianal areas (Figure 1a–d). There were no oral mucosal lesions containing gingivae or palate. Cytology performed using skin lesions showed cocci infection, neutrophilic inflammation and nucleated keratinocytes. Parasitic and fungal skin infections were ruled out by general dermal examination, including skin scrapings, cytology and culture. Differential diagnosis included mucocutaneous pyoderma, mucous membrane pemphigoid (MMP), MCLE and erythema multiforme (EM). Prednisolone previously prescribed was discontinued, and cephalexin (30 mg/kg orally twice daily), ciprofloxacin (20 mg/kg orally once-daily) and mupirocin ointment (once-daily application on lesions) were prescribed to treat bacterial infection and prevent further infections for 10 days before conducting a biopsy procedure.

**Figure 2:**
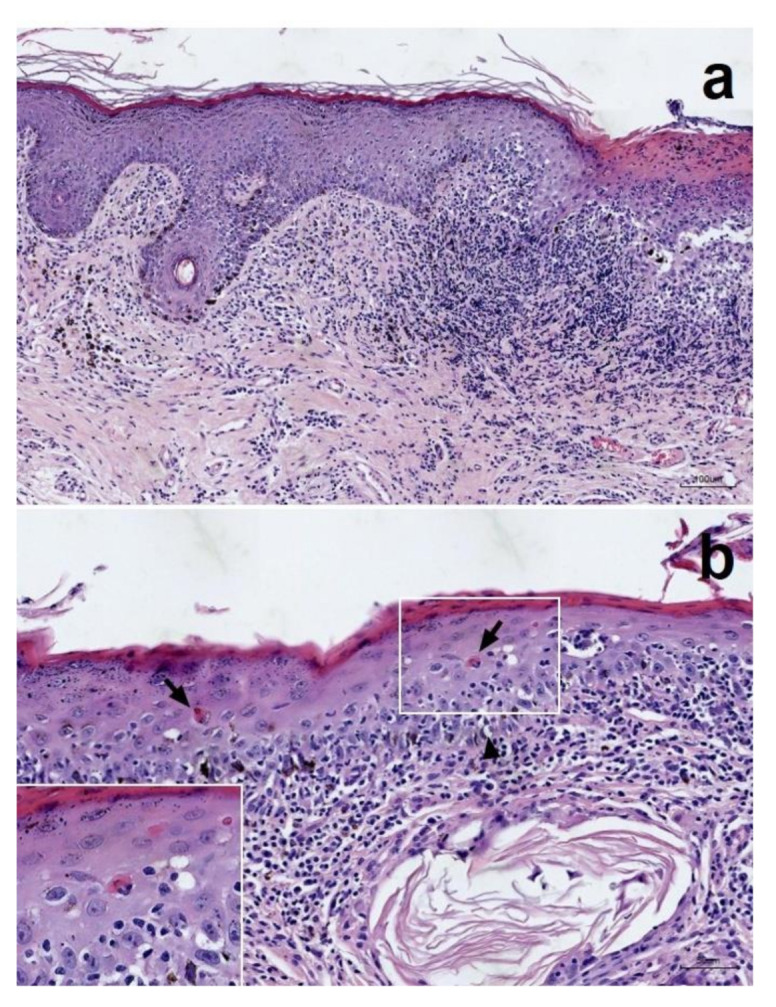
Histopathology of perianal lesions in a dog with MCLE. (**a**) There are mild-to-moderate epidermal hyperplasia, hyperkeratosis and focal severe ulceration with cell-rich lymphocytic interface dermatitis with numerous plasma cells. There is a region of ulceration with a thick layer of cellular crusts. Scale bar represents 100 µm. (**b**) Some basal cell apoptosis with satellitosis was also identified. Scale bar represents 50 µm. Hematoxylin and eosin stain.

**Figure 3:**
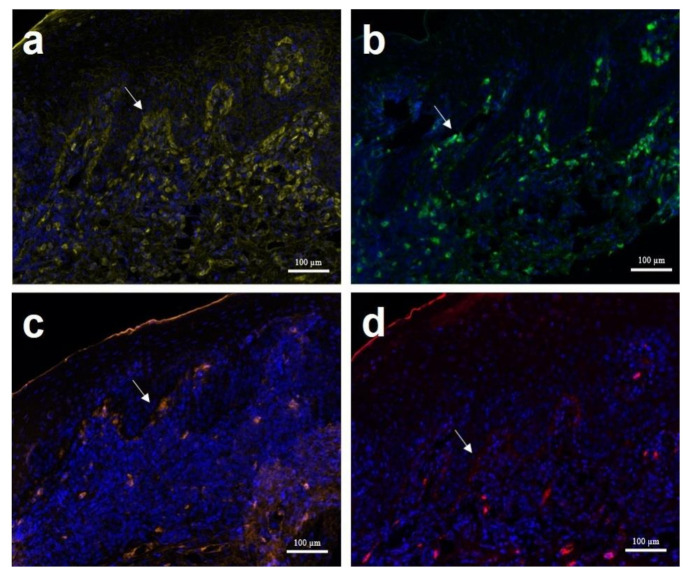
Indirect immunofluorescence of perianal skin in a case of MCLE. Linear deposits of immunoglobulin (Ig)G at the dermoepidermal junction are markedly visible (**a**). In addition, moderate positive lupus band tests of IgA (**b**) and IgM (**c**) and mild positive against C3 (**d**) were also identified. Magnifications, 100×.

Samples from each site of perilabial and peripenial and two sites of perianal skin lesions, all measuring 6 mm in diameter, were collected by punch biopsy. The samples were fixed in 10% neutral buffered formalin and routinely processed and then stained with haematoxylin and eosin, Grocott’s methenamine silver stain (GMS) and periodic acid–Schiff (PAS) stains. Histopathological examination revealed mild-to-moderate epidermal hyperplasia, hyperkeratosis and a region of severe ulceration that was covered by a thick layer of cellular debris, fibrin and rare bacteria. GMS and PAS stains were negative. The underlying dermis was expanded by granulation tissue and suppurative inflammation. In areas with intact epithelium, the dermis contained lymphocytes, plasma cells, neutrophils, macrophages and fewer mast cells. Inflammation multifocally infiltrated the overlying basal cell layer and more superficial layers of the epidermis. Small-to-moderate numbers of apoptotic keratinocytes were identified. There was also evidence of satellitosis. The dermis was oedematous, and there was a background of reactive connective tissue. There was moderate melanin drop-off, and macrophages frequently contained intracytoplasmic pigment (Figure 2). Perianal and peripenial samples were collected for immunohistochemical (IHC) staining. Indirect immunofluorescence (Opal™ automation IHC detection system) performed using fluorescein isothiocyanate-conjugated goat anti-dog immunoglobulin (Ig)G, IgA, IgM and C3 (Novus Biologicals, Littleton, CO, USA) according to the manufacturer’s instructions showed immunoglobulin (Ig)G, IgM, IgA and C3 deposition at the dermoepidermal junction (Figure 3). It revealed strong positive lupus band reaction in the dermoepidermal junction against goat-canine IgG and mild-to-moderate IgA, IgM and C3 deposition. The presence and distribution of skin lesions and histological and immunostaining characteristics were deemed consistent with a diagnosis of MCLE in a previous study [4].

Immediately after the biopsy procedure, treatment was initiated with oral prednisolone (1 mg/kg twice daily), MMF (18.3 mg/kg twice daily), cephalexin (30 mg/kg twice daily) and ciprofloxacin (20 mg/kg once daily) and 0.1% tacrolimus ointment (Protopic; Astellas Pharma GmbH, Munich, Germany) once-daily application. After 2 weeks, the erosion and ulceration lesions were healed, and prednisolone dosage was reduced (0.75 mg/kg twice daily). Complete remission of mucocutaneous lesions was identified on week 4 (Figure 1e–h), and prednisolone dosage was further reduced to 0.5 mg/kg twice daily. The lesions did not worsen even with the dose reduction of prednisolone, and hence it was gradually tapered for an additional 5 weeks and then stopped. Antibiotics were prescribed for a total of 6 weeks, and MMF and 0.1% tacrolimus application were continued for 2 months even after steroid discontinuation. MMF dosage was then tapered to 10 mg/kg twice daily and used in combination with tacrolimus ointment. The dog’s owner returned to the referring hospital at that time, and 6 months after MMF dosage reduction, follow-up via telephone confirmed that the skin condition was being maintained without development of new lesions.

During treatment, the dog had polyuria, polydipsia and polyphagia. Blood analysis and abdominal ultrasonography showed elevated levels of alanine transaminase (133 U/L; reference range 17–78 U/L), alkaline phosphatase (1092 U/L; reference range 47–254 U/L) and gamma-glutamyl transferase (18 U/L; reference range 5–14 U/L) and hepatomegaly. There were no other side effects associated with MMF and tacrolimus treatment.

## 3. Discussion

MCLE, a recently classified variant of CLE, is a rare skin disorder in dogs [4]. Most of the dogs diagnosed with MCLE are purebred, and dogs of large breeds such as German Shepherd and Labrador Retriever are most commonly affected [4]. MCLE was reported in a miniature Pinscher dog, but the incidence and treatment of MCLE in small breeds have still been extremely rarely reported. MMP is common in German Shepherd dogs, and its lesion pattern and location are similar to those of MCLE, which can be confusing during diagnosis [11]. However, MMP more commonly affects the oral cavity, and the lesion spreads beyond the mucosa, accompanied by vesicles and scars, including the characteristic microscopic subepidermal clefts, which distinguish it from MCLE. In the dog in the present case, symmetrical lesions were localized in mucocutaneous areas, including perilabial, peripenial and perianal areas, and no skin or mucosal lesions existed in other areas, including the oral cavity. The lack of systemic signs such as polyarthritis excluded the diagnosis of systemic lupus erythematosus. Analysis of serum antinuclear antibodies (ANAs) was refused due to the financial burden of the owner in this study. A previous study reported that low titres of ANAs were also identified in some of the MCLE cases [4]. While positive ANA serology may be helpful in the diagnosis of SLE, they are not suitable as a tool for differentiation from MCLE. Histopathological features were also different from the typical patterns of MMP. Even minor or major variant of EM may exhibit involvement in the skin mucosa, but EM was excluded due to the dog’s lesion pattern, prominent interface dermatitis and thickening basement membrane. In the tissue of this dog’s perigenital skin, almost the entire structure of the dermoepidermal junction and skin layer were separated due to chronic inflammation, and the dermis was fibrous, thus making it difficult to anticipate a meaningful reading result. In contrast, the perianal biopsy samples demonstrated moderately positive lupus band test results for clear linear deposition at the dermoepidermal basement membrane zone for anti-IgG and moderately positive lupus band test results for anti-IgA and IgM. Positive titers of serum antinuclear antibodies have been reported to be extremely rare [3], and this titer could not be measured due to refusal of the owner in this study.

Previous studies have shown that patients with MCLE respond well to immunosuppressive doses of corticosteroids, with complete remission generally occurring within 1 month [2,4,12]. It has also been reported that tetracycline and niacinamide alone or in combination with corticosteroids may be an effective alternative [4,13,14]. Cyclosporine, a systemic calcineurin inhibitor, has been used alone or in combination with glucocorticoids for the management of several CLEs, and it may be an option to consider when the disease is refractory to other drugs [15,16]. In patients with CLE such as DLE, VCLE and ECLE, the use of cyclosporine showed improvement, but it has been reported that the treatment response was relatively poor in the case of ECLE [15,16,17]. Topical tacrolimus, which is also a calcineurin inhibitor, has been confirmed to be effective in treating autoimmune skin diseases such as DLE and VCLE, although studies are insufficient compared to those on cyclosporine [9,10]. Tacrolimus also exhibited the steroid-sparing effect in the case of subepidermal blistering autoimmune disease, and there have been reports on successful management with oral tetracycline/niacinamide in patients with VCLE [5,7,10]. Previous studies have reported that MMF is more effective and safer than cyclosporine when combined with tacrolimus in human transplant patients [18,19]. At the beginning of treatment of the present case, the combination of systemic prednisolone and MMF and topical tacrolimus induced improvement of mucocutaneous lesions. The previous study reported that immediate recurrence during tapering of drugs, especially corticosteroids, is a limitation that makes it difficult to manage MCLE [3]. Considering the fact that there was no response to previous prescription of anti-inflammatory doses of prednisolone by the referring veterinarian, the authors decided to combine prednisolone with other immunosuppressive drugs due to the possibility of recurrence when prednisolone is reduced to a minimal dose or discontinued. It was confirmed that the combined use of MMF and tacrolimus maintained the complete remission without recurrence for a long period of time even after the complete cessation of corticosteroids. Moreover, there were no side effects associated with both drugs. Although the rapid remission period of less than 1 month represented the effect of prednisolone during the induction period, it is difficult to assume that the effect of immunosuppressive doses of prednisolone lasted for longer than 6 months. However, the effect of prednisolone prescribed at the beginning of treatment in the maintenance period cannot be completely excluded, so further studies are needed on the induction and maintenance effects of the combination of MMF and tacrolimus in MCLE patients. To the best of our knowledge, this is the first report of long-term management of MCLE by treatment with systemic MMF and topical tacrolimus in a dog. However, further studies are required to examine the immunosuppressive effects and safety of the combination of topical tacrolimus and MMF in dogs.

## 4. Conclusions

This case report has demonstrated the clinical and histopathological features of MCLE in a miniature Pinscher dog. The initial treatment was induced using immunosuppressive doses of prednisolone and MMF and tacrolimus ointment, and based on the improvement of the lesion, prednisolone was discontinued after being tapered. When the dosage of MMF was reduced 2 months after prednisolone discontinuation and maintained with tacrolimus ointment, no new lesion development was observed for more than 6 months. No drug-related side effects other than those from the initial high-dose corticosteroids were identified. These results suggest that MMF and tacrolimus can be considered as a safe long-term treatment option and alternatives to previous standard treatments.

## Data Availability

No new data were created or analyzed in this study. Data sharing is not applicable to this article.
